# Correction: Environment-sensitive turn-on fluorescent probes for p53–MDM2 protein–protein interaction

**DOI:** 10.1039/c7md90031g

**Published:** 2017-08-01

**Authors:** Tingting Liu, Yan Jiang, Zhenzhen Liu, Jin Li, Kun Fang, Chunlin Zhuang, Lupei Du, Hao Fang, Chunquan Sheng, Minyong Li

**Affiliations:** a Department of Medicinal Chemistry , Key Laboratory of Chemical Biology (MOE) , School of Pharmacy , Shandong University , Jinan , Shandong 250012 , China . Email: mli@sdu.edu.cn; b Department of Medicinal Chemistry , School of Pharmacy , Second Military Medical University , 325 Guohe Road , Shanghai 200433 , China . Email: shengcq@hotmail.com

## Abstract

Correction for ‘Environment-sensitive turn-on fluorescent probes for p53–MDM2 protein–protein interaction’ by Tingting Liu *et al.*, *MedChemCommun*, 2017, DOI: 10.1039/c7md00287d.



## 

The authors regret an error in Scheme 1, where for probes **9**–**11** it should show *n* = 2–4. The corrected scheme is shown below.

**Scheme 1:**
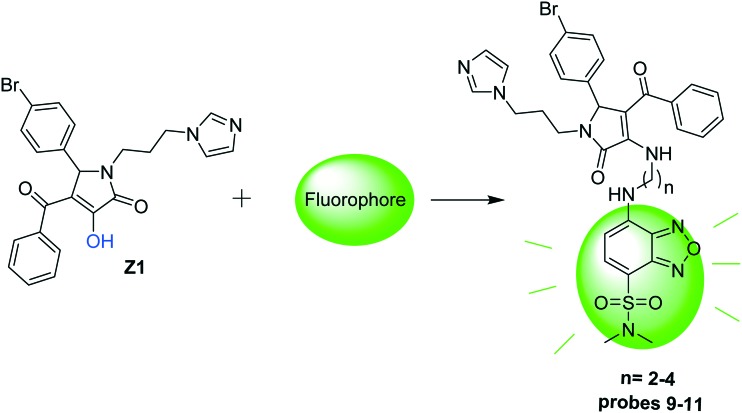


The Royal Society of Chemistry apologises for these errors and any consequent inconvenience to authors and readers.

